# Supplementary material for: MetaBoot: a machine learning framework of taxonomical biomarker discovery for different microbial communities based on metagenomic data
Source: PeerJ. 2015 Jul 7;3:e993. doi: 10.7717/peerj.993 (PMC4512773; doi:10.7717/peerj.993)
Supplement: Supplemental Information 1 — This file includes four supplemental figures. [file peerj-03-993-s001.docx]

**Supplementary information for “MetaBoot: A machine learning framework of taxonomical** **biomarker discovery for different microbial communities based on metagenomic data”**

**Xiaojun Wang^1,2^, Wei Fang^1,4^, Xiaoquan Su^1,3^, Xinping Cui^1,5,^*, Kang Ning^1,2,^***

^1^ Bioinformatics Group of Single Cell Center, Shandong Key Laboratory of Energy Genetics and CAS Key Laboratory of Biofuels, Qingdao Institute of Bioenergy and Bioprocess Technology, Chinese Academy of Sciences, Qingdao 266101, Shandong Province, People’s Republic of China

^2^ University of Chinese Academy of Sciences, Beijing 100864, People’s Republic of China

^3^ CUDA Reserach Center, Qingdao Institute of Bioenergy and Bioprocess Technology, Chinese Academy of Sciences, Qingdao 266101, Shandong Province, People’s Republic of China

^4^ Center for Bioinformatics, College of Life Science, Northwest A & F University, Yangling 712100, Xi’an City, Shaanxi Province, People’s Republic of China

^5^ Department of statistics, University of California, Riverside. Riverside, California, USA

*Corresponding author:

E-mail: ningkang@qibebt.ac.cn

xinping.cui@ucr.edu

Running title: Biomarker discovery for microbial communities.

Email-address for all authors:

Xiaojun Wang: wang_xj@qibebt.ac.cn

Wei Fang: fangwei@qibebt.ac.cn

Xiaoquan Su: suxq@qibebt.ac.cn

Xinping Cui: xinping.cui@ucr.edu

Kang Ning: ningkang@qibebt.ac.cn

**Contents**

[Details for selecting *M* and *B* for MetaBoot analysis of synthetic data *S2* and *S3* 3](#_Toc394219884)

[Details for Results on synthetic dataset 4](#_Toc394219885)

[Details for Results on oral dataset 7](#_Toc394219886)

[References 8](#_Toc394219887)

# Details for selecting *M* and *B* for MetaBoot analysis of synthetic data *S2* and *S3*

**
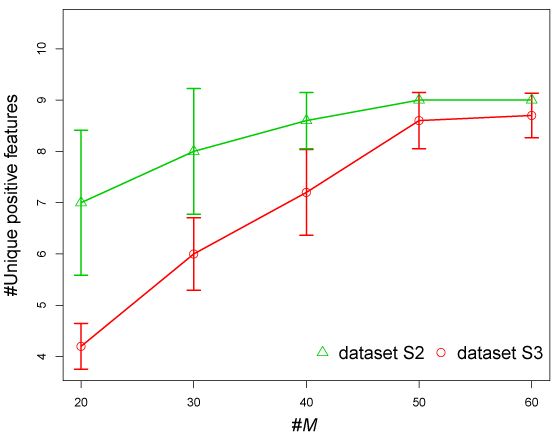
**

**(a)**

**
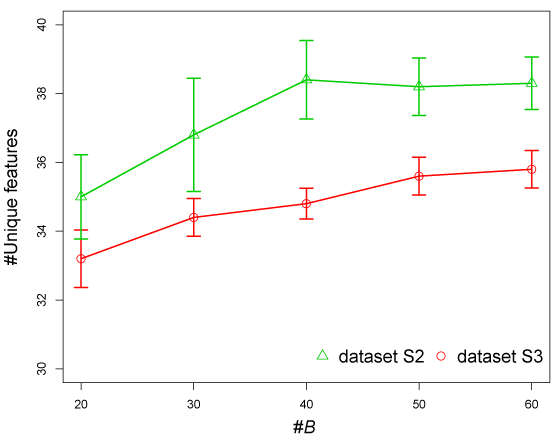
**

**(b)**

**Figure S1.** **The plots for selecting *M* and *B* for MetaBoot analysis of synthetic data *S2* and *S3*. (a)** The x axis is the values of *M* (The number of features to be selected in the first feature selection step). And the y axis is the number of unique positive features selected by mRMR for each given *M*. **(b)** The x axis is the number of bootstraps B. The y axis is the number of unique features selected by all bootstrap processes.

The MetaBoot analysis process includes 3 major steps (first feature selection step, bootstrap and feature selection step, and feature rank step). *M* represents the number of features selected in the first feature selection step. *B* represents the number of bootstrap process in bootstrap and feature selection step. As shown in **Figure S1**, for synthetic dataset *S2* and *S3,* the parameters *M* and *B* were set to 50 and 40, respectively.

# Details for Results on synthetic dataset

**
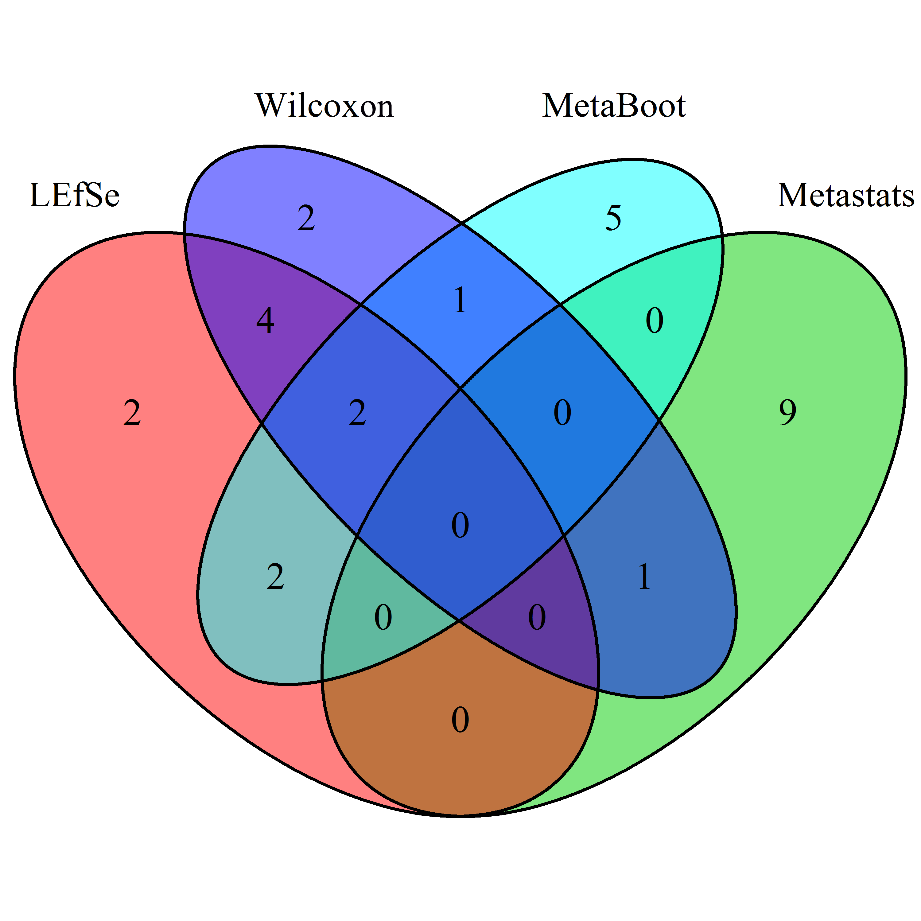
**

**(a)**

**
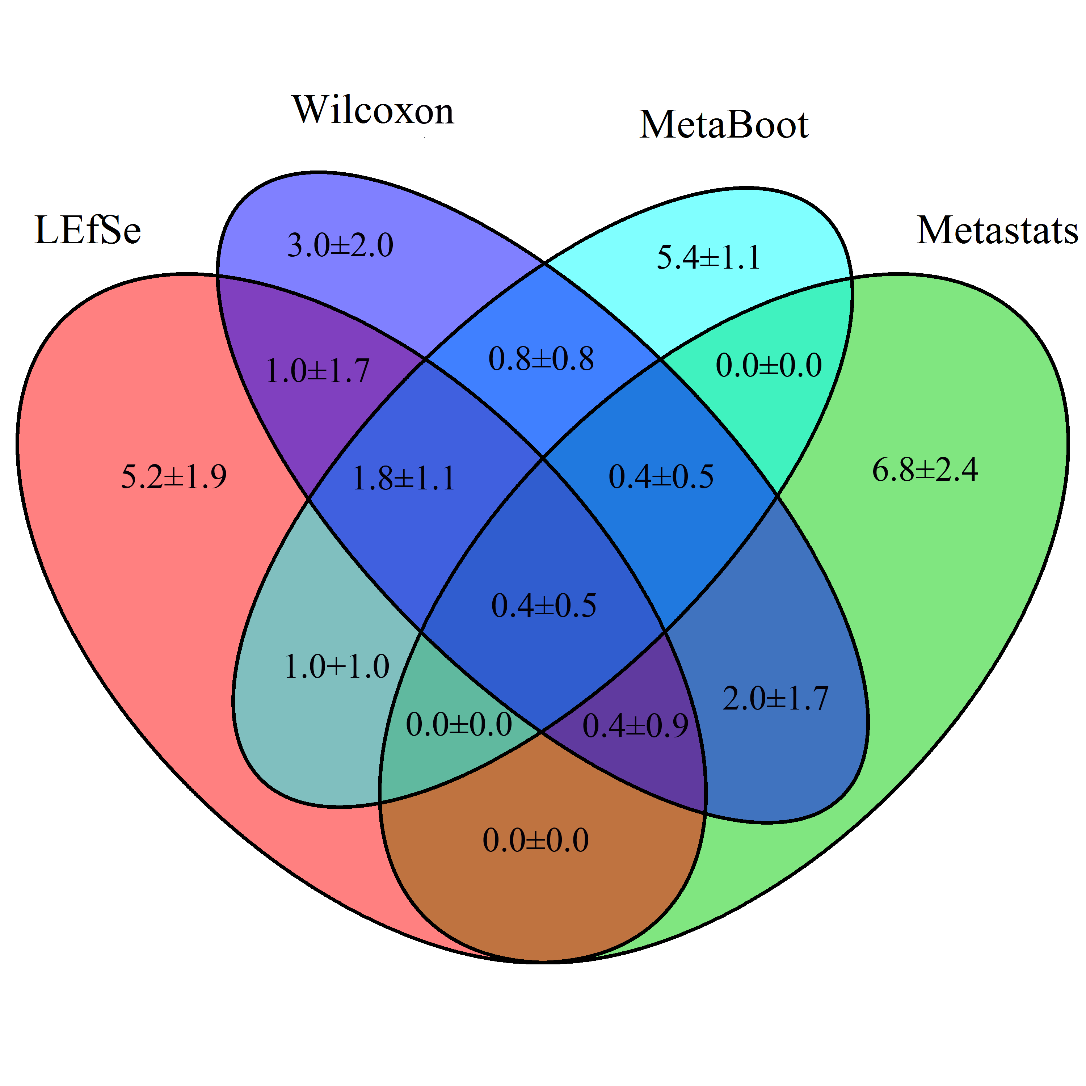
**

**(b)**

**Figure S2. Results on synthetic dataset *S1*. (a)** The Venn diagram for 10 features selected from synthetic dataset *S1* (*sd*=1) using these 4 methods. **(b)** The Venn diagram for 10 features selected using these 4 methods.


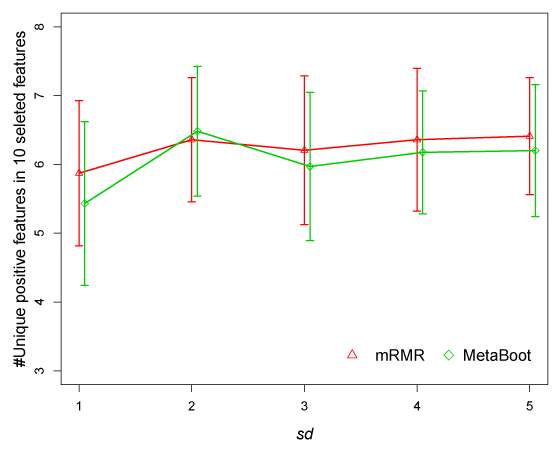


**(a)**


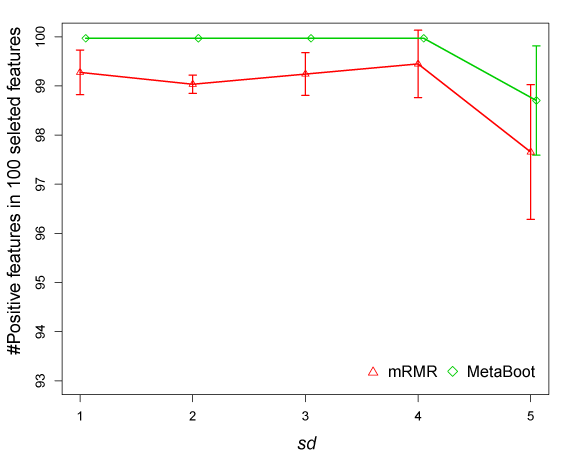


**(b)**

**Figure S3.** **Comparison of results by mRMR and MetaBoot for synthetic dataset *S1* in selecting (unique) positive features.** The x axis is the standard deviation (*sd*) representing the parameter *sd*s in synthetic dataset *S1*. The y axis is the number of positive features in 100 selected features. The error bar represents 1 standard deviation.


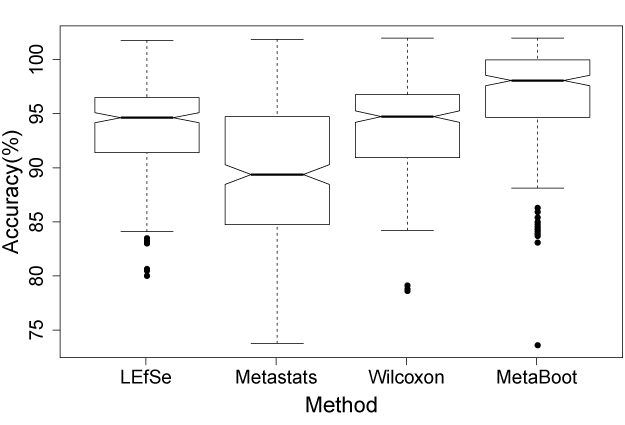


**Figure S4**. **Comparison of accuracies when using 10 features selected by 4 methods based on synthetic dataset *S2*.** The x axis represents 4 methods and the y axis represented classification accuracy by SVM.

# Details for Results on oral dataset


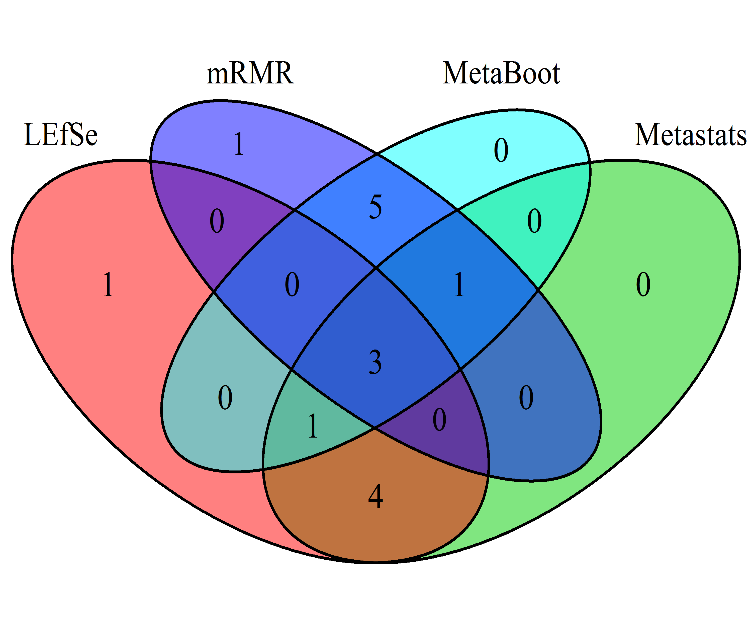


**(a)**


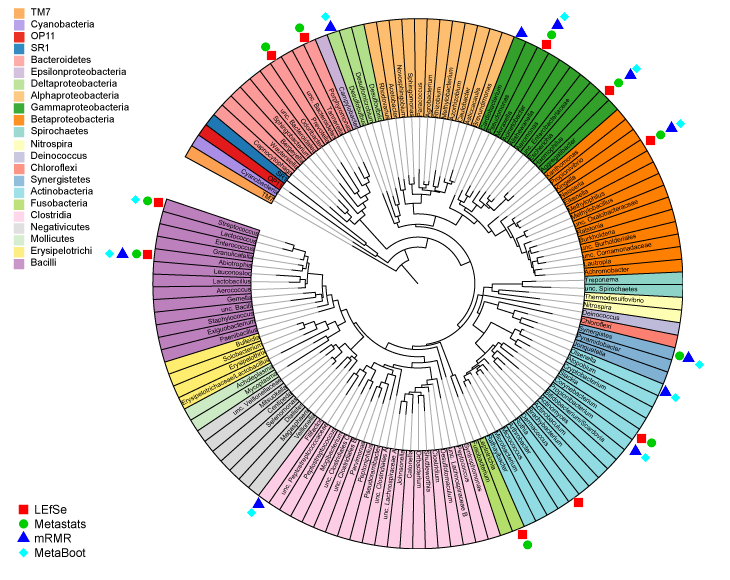


**(b)**

**Figure S5.** **Results on oral dataset.** **(a)** The Venn diagram when we selected 10 features from oral dataset using these 4 methods. **(b)** Circular phylogenetic tree of oral dataset at level of genus. The tree was generated with RAxML and viewed in ITOL[1]. Genera are color-coded by phyla, except for the Firmicutes and Proteobacteria, which are shown at the level of class. We used the same phylogenetic tree plot from microbiome.osu.edu[2], and we added legends onto this tree to show biomarkers selected by different methods.

# References

1. Letunic I, Bork P (2007) Interactive Tree Of Life (iTOL): an online tool for phylogenetic tree display and annotation. Bioinformatics 23: 127-128.

2. Griffen AL, Beall CJ, Firestone ND, Gross EL, DiFranco JM, et al. (2011) CORE: a phylogenetically-curated 16S rDNA database of the core oral microbiome. PloS one 6: e19051.
